# Salvage radiation therapy in prostate cancer: relationship between rectal dose and long-term, self-reported rectal bleeding

**DOI:** 10.1007/s12094-020-02433-4

**Published:** 2020-07-03

**Authors:** K. Braide, J. Kindblom, U. Lindencrona, J. Hugosson, N. Pettersson

**Affiliations:** 1 Department of Urology, Institute of Clinical Sciences, University of Gothenburg, Gothenburg, Sweden; 2 Department of Urology, Sahlgrenska University Hospital, Gothenburg, Sweden; 3 Department of Oncology, Institute of Clinical Sciences, University of Gothenburg, Gothenburg, Sweden; 4 Department of Oncology, Sahlgrenska University Hospital, Gothenburg, Sweden; 5 Department of Radiation Physics, Institute of Clinical Sciences, The Sahlgrenska Academy, University of Gothenburg, Gothenburg, Sweden; 6 Department of Medical Physics and Biomedical Engineering, Sahlgrenska University Hospital, Gothenburg, Sweden

**Keywords:** Prostate cancer, Salvage radiation therapy, Self-reported side-effects, Rectal dose and rectal bleeding

## Abstract

**Purpose:**

To quantify the relationship between the rectal dose distribution and the prevalence of self-reported rectal bleeding among men treated with salvage radiotherapy (ST) delivered by three-dimensional conformal radiotherapy (3DCRT) for prostate cancer. To use this relationship to estimate the risk of rectal bleeding for a contemporary cohort of patients treated with volumetric modulated arc therapy (VMAT) ST.

**Methods and patients:**

Rectal bleeding of any grade was reported by 56 (22%) of 255 men in a PROM-survey at a median follow-up of 6.7 years after 3DCRT ST. Treatment plan data were extracted and dose–response relationships for the rectal volumes receiving at least 35 Gy (V_35Gy_) or 63 Gy (V_63Gy_) were calculated with logistic regression. These relationships were used to estimate the risk of rectal bleeding for a cohort of 253 patients treated with VMAT ST.

**Results:**

In the dose–response analysis of patients in the 3DCRT ST cohort, both rectal V_35Gy_ and V_63Gy_ were statistically significant parameters in univariable analysis (*p* = 0.005 and 0.003, respectively). For the dose–response models using either rectal V_35Gy_ or V_63Gy_, the average calculated risk of rectal bleeding was 14% among men treated with VMAT ST compared to a reported prevalence of 22% for men treated with 3DCRT ST.

**Conclusions:**

We identified dose–response relationships between the rectal dose distribution and the risk of self-reported rectal bleeding of any grade in a long-term perspective for men treated with 3DCRT ST. Furthermore, VMAT ST may have the potential to decrease the prevalence of late rectal bleeding.

## Introduction

Rectal bleeding as a side-effect after prostate cancer radiation therapy, PCRT, is frequently reported and the extent has been shown to be related to absorbed dose to the rectum [1, 2]. However, most data are from primary PCRT while there is limited information on the risk of rectal bleeding in the postoperative setting, delivered by salvage radiation therapy, ST [3, 4]. Since ST is a rescue after radical prostatectomy in prostate cancer and the only curative treatment option when there is a PSA-relapse after surgery, knowledge of curability and side-effects seem crucial. As many as 30–40% of the patients treated for prostate cancer with surgery will suffer from PSA-relapse [5–7] and will probably be presented the option on ST.

In a recent study in our department, we investigated patient-reported outcomes after ST. A cohort of men, treated with 3DCRT during 2005–2010, was invited and 255/325 adhered the invitation and shared their experiences on side-effects (manuscript accepted, 10.1080/21681805.2020.1782980) in a cross-sectional survey on Patient Reported Outcome Measures (PROM). A group of men matched according to age, year of surgery, and hospital, that had gone through only radical prostatectomy (RP) served as a control group. The median time from surgery to survey was 10 years. For the men treated with ST, the median time from ST to survey was 6.7 years. In the ST group, symptoms from the rectum with bleeding and leakage were dominating; almost every fourth patient reported any bleeding at the time of the survey. To help overcome the lack of data in the postoperative setting [8], we investigated the relationship between rectal irradiation and patient-reported late rectal bleeding in the 3DCRT ST setting. Furthermore, since many patients today are treated by volumetric modulated arc therapy (VMAT) we also investigated how these results could be applied in the VMAT ST setting.

The aim of this work was to quantify the relationship between the rectal dose distribution and the prevalence of self-reported rectal bleeding among men treated with 3DCRT ST for prostate cancer. This relationship was then used to estimate the risk of rectal bleeding for a contemporary cohort of patients treated with VMAT ST.

## Methods and patients

Two cohorts of men treated with ST following radical prostatectomy at the Sahlgrenska University Hospital, Göteborg, Sweden were investigated in this study. The Research Ethics Board at Gothenburg University Hospital approved the study (EPN 488-13). The first cohort, Group 1, consisted of all men that underwent 3DCRT ST during 2005–2010, still alive (*n* = 324). They were invited to participate in a study on treatment-related morbidity after RT. The survey was given as a questionnaire to be filled out at home and then returned to the office. The survey is used nationwide in Sweden and data are available through the National Prostate Cancer Register (NPCR, www.npcr.se, [9, 10]. In short, 255 (79%) out of 324 men filled out and returned the questionnaire. Fifty-six (22%) out of 255 reported any grade (‘a little’, ‘to some extent’,’very much’) of rectal bleeding according to the question ‘Do you have blood in your stools?’ The median time to follow-up was 6.7 years [range 5.3–8.2]. The second cohort, Group 2, were all patients treated with VMAT ST during 2017–2018 (*n* = 253). For the patients in Group 2, follow-up data on rectal bleeding were not available.

### Treatment procedure and collection of treatment plan data

In Group 1, pre-treatment CT imaging with a slice thickness of 2.5–5 mm were acquired for treatment planning. In Group 2, pre-treatment CT imaging with a slice thickness of 2–3 mm were acquired. Patient instructions at CT imaging were to empty the rectum and to keep a comfortably filled bladder. For the treatment sessions, no instructions on rectal emptying was given but the bladder instruction was the same as for the CT imaging. In Group 1, instructions for CTV delineation varied according to the physician’s discretion but from 2007 the EORTC guidelines were gradually introduced [11]. In Group 2, the CTV was mostly delineated according to the RTOG guidelines [12]. In Group 1, the CTV-to-PTV margin was 10 or 15 mm in all directions while it was 10 mm in all directions for patients in Group 2. The rectum and the bladder were considered organs-at-risk and were delineated, including filling, for all patients. The instruction for rectum delineation was to contour from the anal canal to the sigmoidal-rectum inflection point or to 5 cm superiorly of the midpoint of the target, whichever occurred first.

For the patients in Group 1, 248 out of 255 treatment plans were created using a 3DCRT technique with typically one anterior–posterior field and two opposed lateral fields (Eclipse, Varian Medical Systems, Palo Alto, CA, US). All fields had a photon beam energy of 15 MV and were individually shaped with a multi-leaf collimator (MLC) and wedges were used, whenever judged to be appropriate. Seven treatment plans in Group 1 were 7-field IMRT plans with a photon beam energy of 6 MV. For the patients in Group 2, all 253 treatment plans were created using a VMAT technique with two 360-degree arcs each using a photon beam energy of 6 MV.

**Fig. 1:**
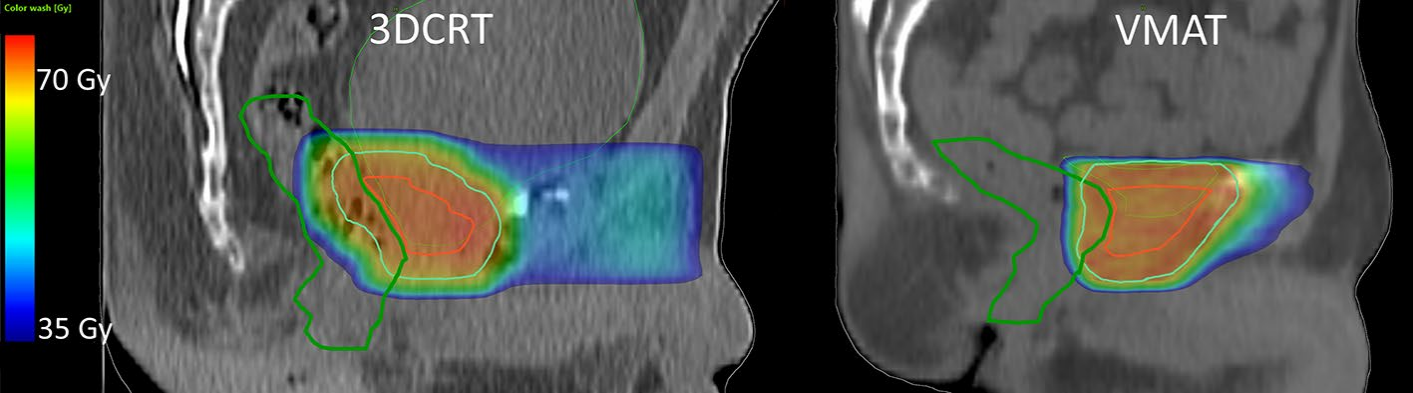
Sagittal view of one typical treatment plan for three-dimensional conformal radiotherapy (3DCRT, left-hand side) from Group 1 and of one for volumetric modulated arc therapy (VMAT, right-hand side) from Group 2. The delineated rectums are shown in dark green, the clinical target volumes (CTVs) in red and the planning target volumes (PTVs) in cyan

The prescribed dose was in all cases 70 Gy in 2-Gy fractions (35 × 2.0 Gy). Treatment plan objectives included that the PTV should be covered by the 95% isodose and that the CTV mean dose should be 70 Gy. For patients in Group 1, the planning instructions were to minimize the rectal dose. In Group 2, the rectal treatment planning objectives were to keep the relative rectal volume receiving at least 35 Gy below 50% (V_35Gy_ < 50%) and that the relative rectal volume receiving at least 63 Gy (V_63Gy_) should be minimized. The dose levels of 35 and 63 Gy correspond to 50 and 90% of the 70-Gy prescription dose, respectively. Figure 1 shows a sagittal view of one 3DCRT ST treatment plan and of one VMAT ST treatment plan.

The treatment was delivered by daily 2-Gy fractions for 7 weeks. In Group 1, laser-based setup followed by orthogonal kilovoltage (kV) or megavoltage (MV) imaging was done for the first four fractions. For these four fractions, the treatment couch was moved according to the bony anatomy match and in the subsequent fractions, the patient was set up according to the average of the first four couch positions. One additional acquisition of orthogonal kV or MV images per week was then used to confirm the used setup. In Group 2, patients were imaged by daily orthogonal kV imaging and positioned according to bony anatomy.

### Data collection and statistical analysis

For all patients in both groups, we extracted the rectal volumes, the relative rectal V_35Gy_ and V_63Gy_, and the PTV volumes using the Eclipse scripting functionality. Rectal V_35Gy_ and V_63Gy_ were chosen since these are our clinically used treatment plan objectives, and they are in accordance with previously investigated rectal volume parameters [13, 14].

For patients in Group 1, we performed dose–response analysis to quantify the impact of rectal dose on the risk of rectal bleeding. Each patient contributed with an endpoint of either 0 (no rectal bleeding) or 1 (rectal bleeding). Relative rectal V_35Gy_ and V_63Gy_ were separately investigated in univariable logistic regression using the maximum likelihood estimation method. No multivariable analysis was performed. Statistical significance for the models were assessed with the likelihood ratio test and a *p* value below 0.05 was considered statistically significant. Confidence intervals (CIs) for the dose–response curves were estimated with bootstrapping using 5000 bootstrap samples.

The resulting dose–response relationships were used to calculate the probability of rectal bleeding for each patient in both groups. Welch’s *t* test (Student’s unpaired *t* test without assuming equal variances) was used to assess differences between Group 1 and 2 for relative rectal V_35Gy_, relative rectal V_63_ Gy, and the calculated probabilities. Group-wise differences between rectal volumes, V_35Gy_ and V_63Gy_ were also assessed with Welch’s *t* test. The 95% CIs for average differences between groups were calculated. The relationship between the PTV volume and rectal dose (V_35Gy_ and V_63Gy_) was assessed using linear regression. All statistical analysis was performed in Matlab (version 2019b, The MathWorks Inc., Natick, MA, US).

## Results

**Fig. 2:**
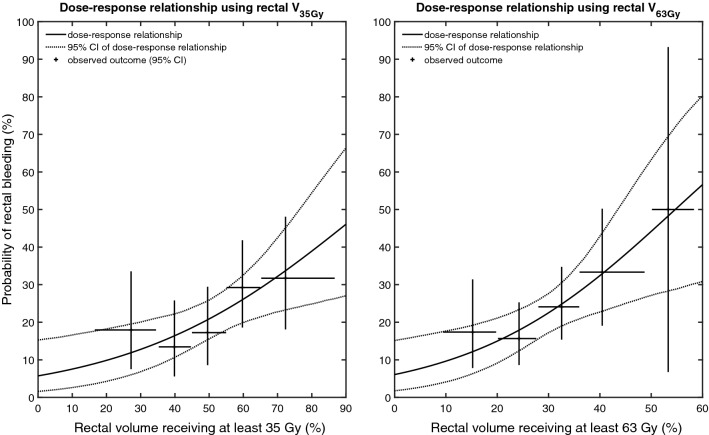
Dose–response relationships for self-reported rectal bleeding of any grade. Left-hand side panel: the relationship between the relative rectal volume receiving at least 35 Gy (V_35Gy_) and rectal bleeding; right-hand side panel: the relationship between the relative rectal volume receiving at least 63 Gy (V_63Gy_) and rectal bleeding. Both panels: The solid black lines are the estimated relationships between the rectal dose and the probability of rectal bleeding, and the dashed black curves are the 95% confidence intervals (CIs). The observed data have been stratified into five bins to illustrate the model fit. The horizontal bars show the range for each bin and the vertical bars are the 95% CIs for the observed prevalence of rectal bleeding

In the dose–response analysis of self-reported rectal bleeding of any grade for the 255 patients in Group 1, both relative rectal V_35Gy_ and V_63Gy_ were statistically significant parameters in univariable analysis (*p* = 0.005 and *p* = 0.003, respectively). The estimated dose–response relationships and their 95% CIs are shown in Fig. 2.

**Fig. 3:**
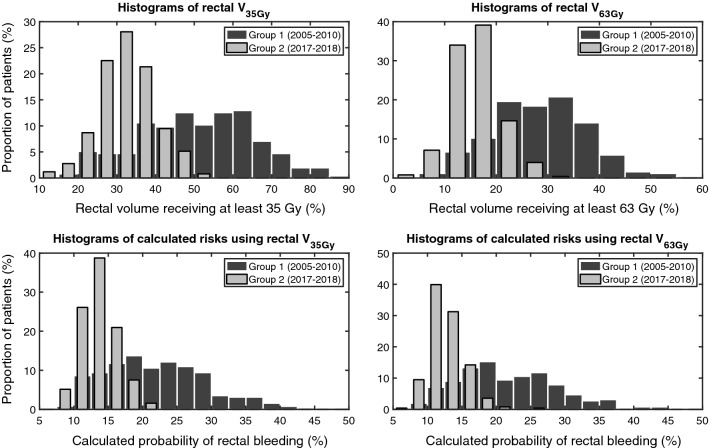
Upper panels: Histograms of the distributions of the relative rectal volume receiving at least 35 Gy (V35Gy, left-hand side) and relative rectal volume receiving at least 63 Gy (V63Gy, right-hand side). Lower panels: Histograms of the calculated risks of rectal bleeding for the dose–response model using rectal V35Gy as a parameter (left-hand side) and using rectal V65Gy as a parameter (right-hand side). In each panel, data are shown separately for Group 1 and Group 2

The distribution of relative rectal V_35Gy_ and V_63Gy_ for both groups is given in the top row of Fig. 3. The average ± 1 standard deviation (SD) rectal V_35Gy_ was 50.4 ± 14.8% for patients in Group 1 and 32.8 ± 7.4% for patients in Group 2. The average V_35Gy_ in Group 1 was 17.6 percentage points larger (95% CI 15.6–19.7) than in Group 2. The average rectal V_63Gy_ in Group 1 was 28.3 ± 9.0% and 16.0 ± 4.5% in Group 2. The average V_63Gy_ in Group 1 was 12.3 percentage points larger (95% CI 11.0–13.5) than in Group 2.

Calculated risks of rectal bleeding (applying the dose–response relationships in Fig. 2 using the V_35Gy_ and V_63Gy_ in the top row of Fig. 2) are shown in the bottom row of Fig. 3. For the dose–response model using V_35Gy_ as a parameter, the average calculated risk in Group 1 was 22.0 ± 7.3% and 13.9 ± 2.6% in Group 2. The average calculated risk in Group 1 was 8.0 percentage points larger (95% CI 7.1–9.0) than in Group 2. For the dose–response model using V_63Gy_ as a parameter, the average calculated risk in Group 1 was 22.0 ± 7.7% and 12.8 ± 2.6% in Group 2. The average calculated risk in Group 1 was 9.2% points larger (95% CI 8.2–10.2) than in Group 2.

**Fig. 4:**
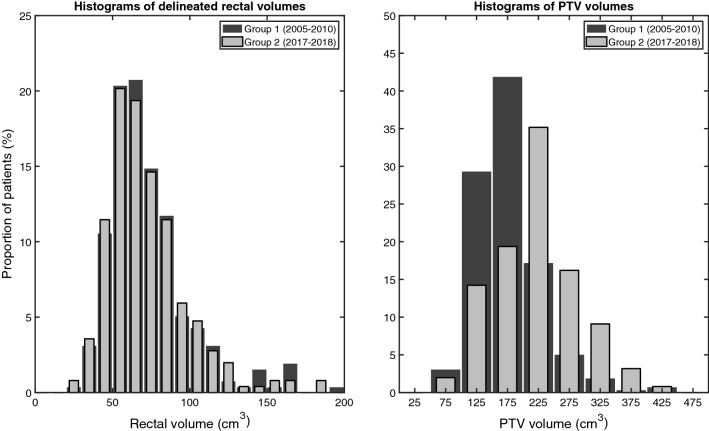
Histograms of the distributions of delineated rectal volume (left-hand side) and the volume of the planning target volume (PTV, right-hand side). In each panel, data are shown separately for Group 1 and Group 2

Distributions of rectal and PTV volumes are given in Fig. 4. The average delineated rectal volume was 73.5 ± 26.6 cm^3^ in Group 1 and 72.6 ± 26.2 cm^3^ in Group 2. The average delineated rectal volumes in Group 1 and Group 2 were similar with an average difference of 0.9 cm^3^ (95% CI − 3.7–5.5 cm^3^). The average PTV volume was 177 ± 53 cm^3^ in Group 1 and 221 ± 67 cm^3^ in Group 2. The PTV volume in Group 2 was on average 43.2 cm^3^ larger (95% CI 32.6–53.8) than in Group 1.

**Fig. 5:**
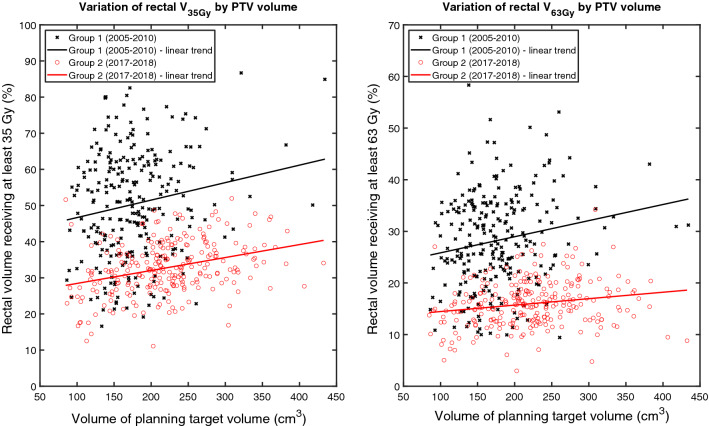
Left-hand side: The variation of the relative rectal volume receiving at least 35 Gy (V35Gy) by the volume of the planning target volume (PTV). Right-hand side: Variation of the relative rectal volume receiving at least 63 Gy (V63Gy) by PTV volume. In each panel, data are stratified according to Group 1 and Group 2. The linear trend for each set of data is shown as a solid line

The variation of rectal V_35Gy_ and V_63Gy_ by PTV volume for each group is given in Fig. 5. Within each group, average rectal V_35Gy_ and rectal V_63Gy_ were affected by the size of the PTV whereby larger PTV volumes were associated with higher average V_35Gy_ and V_63Gy_. Increasing the PTV volume in Group 1 and Group 2 by 100 cm^3^ increases the V_35Gy_ by on average 4.8 and 3.1% points, respectively. Similarly, increasing the PTV volume in Group 1 and Group 2 by 100 cm^3^ increases the V_63Gy_ by on average 3.1 and 1.2% points, respectively.

## Discussion

In this study, we found relationships between absorbed dose to the rectum and the risk of rectal bleeding of any grade among 255 men treated with 3DCRT ST. Rectal bleeding was assessed through a PROM survey administered in median 6.7 years after 3DCRT ST. Rectal V_35Gy_ and V_63Gy_ were both found to impact the risk of rectal bleeding and their relation to the probability of rectal bleeding was quantified by dose–response relationships. The observed prevalence of any rectal bleeding for the patients treated with 3DCRT ST (Group 1) was 22% (56/255), and using our estimated dose–response relationships, the average calculated risk in the VMAT cohort (Group 2) was about 14%.

This is, to the best of our knowledge, the first study to have quantified relationships between rectal dose and the risk of patient-reported rectal bleeding among men treated by 3DCRT ST. Rectal bleeding, using mostly objective/clinician-reported endpoint definitions, has previously been studied [13, 14]. Our results are in accordance with those previous studies in that larger irradiated rectal volumes to medium–high (35 Gy) and high (63 Gy) doses resulted in higher rates of rectal bleeding. The relationships in Fig. 2 can be used to derive treatment plan objectives for future patients. For example, if a maximum risk for rectal bleeding of 15% is deemed acceptable, rectal V_35Gy_ should be kept below 36% and rectal V_63Gy_ below 20%.

The assessment of rectal bleeding can be through PROM data or objective/clinician-reported [15, 16] and the latter is the most utilized but is reported to underestimate the symptom rates [17, 18]. In our study, we utilized a PROM survey which we consider will result in more clinically relevant estimates of symptom rates. Still, maybe the best evaluation of rectal symptoms is a combination of the two, and, to also keep in mind the possibility that there are other rectal disorders that can cause the bleeding, not only depending on the delivered radiation treatment [1, 19]. In our cohort, there was, for instance, one patient with rectal bleeding and concomitant warfarin medication. In our PROM survey, there was also a control group, that had gone through surgery only (*n* = 485), and their response to the survey revealed a mere 3% prevalence of rectal bleeding of any grade compared to 22% in the 3DCRT ST group. This means that the majority of reported rectal bleeding most likely originates from the RT.

We estimated the potential benefit of VMAT ST to decrease the prevalence of rectal bleeding using rectal dose data from a contemporary cohort of patients treated at our institution. In this VMAT group, we found the calculated risk for rectal bleeding to be 13–14% which was considerably lower than the 22% observed prevalence of rectal bleeding in the 3DCRT group. However, the reaction to irradiation of the rectum occurs in the rectal wall, not in its content, and will cause inflammation in the mucosa and develop telangiectasias with time [20] which would be a major reason for rectal bleeding. Gomez et al., investigated the differences between contouring the whole rectum and the rectal wall in prostate cancer treatment with intensity-modulated RT (IMRT) [21]. They showed that larger relative volumes of the rectal wall were irradiated than the corresponding solid organs in the high-dose area. Modern RT techniques such as IMRT and VMAT may not spare the rectal wall as much as indicated by the DVH for the entire rectal volume. This supposed lack of rectal wall sparing could be one potential reason that modern prostate RT techniques do not always result in less rectal toxicity compared to 3DCRT [22–24]. Our calculated risk reduction is currently only theoretical, and future studies are necessary to evaluate to what extent VMAT can assist in reducing rectal bleeding in the postoperative setting.

Comparing the 3DCRT and VMAT groups, the calculated risk reduction in the VMAT group remained even though the changes in target definition rendered bigger PTVs in this group. To note is that our delineation of CTV has changed over time and in the 3DCRT group the EORTC guideline [11] was used from 2007 and further on. Before 2007 the delineation was, in some way varying and depending on the physicians’ earlier experience. In the VMAT group, most delineations were corresponding to the RTOG guideline [12] which resulted in larger PTVs including more rectal volume in the treatment plans [25]. However, the instructions for delineation of the rectum in our two groups have been the same over the years, and this has resulted in very similar rectal volumes (Fig. 3).

Another important issue regarding the development of rectal toxicity in prostate cancer RT is the setup procedure accuracy and rectal daily variation which influence differences between treatment plan rectal dose and the patient rectal dose at treatment delivery. Our own clinical experience, and others reporting [26], include observing substantial variations of rectal volume and location during a 7-week treatment delivery period, which can influence the exposure of the rectal wall to irradiation through position changes. These position differences during the treatment period has been discussed by other authors as leading to under-and-overdosage to CTV and OARs [27, 28]. Using hydrogel tissue fiducial marker for patient setup in the postoperative setting is an emerging technique [29] that potentially could decrease the rectum dose variation. In our VMAT series there was a daily matching procedure using kV imaging, but to skeletal structures, compared to the 3CDRT group where the matching was performed in a similar way but on a weekly basis. One proposed way to handle these changes is through the patient setup using cone-beam CT (CBCT) [30, 31]. CBCT matching is now under development in our department. We do however believe that the inter-fractional rectal variation has a limited impact on the validity of the dose–response relationships in this study (as in most studies of rectal toxicity) since the treatment plan rectal DVH has captured the main feature of delivered dose distribution. Furthermore, all patients treated by VMAT are also treated with the same overall 7-week treatment time and their rectal DVHs are therefore similarly impacted by inter-fractional rectal variations.

## Conclusion

In this study, we identified dose–response relationships between the rectal dose distribution and the risk of self-reported rectal bleeding of any grade in a long-term perspective for men treated with 3DCRT ST. Furthermore, the calculated average risk of rectal bleeding for a contemporary cohort of patients treated with VMAT ST was lower than for the patients in the 3DCRT cohort.

## References

[1] Michalski JM, Gay H, Jackson A, Tucker SL, Deasy JO (2010). Radiation dose-volume effects in radiation-induced rectal injury. Int J Radiat Oncol Biol Phys.

[2] Jadon R, Higgins E, Hanna L, Evans M, Coles B, Staffurth J (2019). A systematic review of dose-volume predictors and constraints for late bowel toxicity following pelvic radiotherapy. Radiat Oncol.

[3] Akthar AS, Wong AC, Parekh AD, Hubert G, Son CH, Pelizzari CA (2018). Late toxicity after post-prostatectomy intensity modulated radiation therapy: evaluating normal-tissue sparing guidelines. Adv Radiat Oncol.

[4] Ingrosso G, Carosi A, di Cristino D, Ponti E, Lancia A, Murgia A (2017). Volumetric image-guided highly conformal radiotherapy of the prostate bed: toxicity analysis. Rep Pract Oncol Radiother.

[5] Bianco FJ, Scardino PT, Eastham JA (2005). Radical prostatectomy: long-term cancer control and recovery of sexual and urinary function ("trifecta"). Urology.

[6] Boorjian SA, Thompson RH, Tollefson MK, Rangel LJ, Bergstralh EJ, Blute ML (2011). Long-term risk of clinical progression after biochemical recurrence following radical prostatectomy: the impact of time from surgery to recurrence. Eur Urol.

[7] Andersen S, Richardsen E, Nordby Y, Ness N, Storkersen O, Al-Shibli K (2014). Disease-specific outcomes of radical prostatectomies in Northern Norway; a case for the impact of perineural infiltration and postoperative PSA-doubling time. BMC Urol.

[8] Raziee H, Berlin A (2016). Gaps between evidence and practice in postoperative radiotherapy for prostate cancer: focus on toxicities and the effects on health-related quality of life. Front Oncol.

[9] Widmark A, Fransson P, Tavelin B (1994). Self-assessment questionnaire for evaluating urinary and intestinal late side effects after pelvic radiotherapy in patients with prostate cancer compared with an age-matched control population. Cancer.

[10] Fransson P, Tavelin B, Widmark A (2001). Reliability and responsiveness of a prostate cancer questionnaire for radiotherapy-induced side effects. Support Care Cancer.

[11] Poortmans P, Bossi A, Vandeputte K, Bosset M, Miralbell R, Maingon P (2007). Guidelines for target volume definition in post-operative radiotherapy for prostate cancer, on behalf of the EORTC Radiation Oncology Group. Radiother Oncol.

[12] Michalski JM, Lawton C, El Naqa I, Ritter M, O'Meara E, Seider MJ (2010). Development of RTOG consensus guidelines for the definition of the clinical target volume for postoperative conformal radiation therapy for prostate cancer. Int J Radiat Oncol Biol Phys.

[13] Olsson CE, Jackson A, Deasy JO, Thor M (2018). A systematic post-QUANTEC review of tolerance doses for late toxicity after prostate cancer radiation therapy. Int J Radiat Oncol Biol Phys.

[14] Thor M, Apte A, Deasy JO, Karlsdottir A, Moiseenko V, Liu M (2013). Dose/volume-response relations for rectal morbidity using planned and simulated motion-inclusive dose distributions. Radiother Oncol.

[15] Cox JD, Stetz J, Pajak TF (1995). Toxicity criteria of the Radiation Therapy Oncology Group (RTOG) and the European Organization for Research and Treatment of Cancer (EORTC). Int J Radiat Oncol Biol Phys.

[16] Einstein DJ, Patil D, Chipman J, Regan MM, Davis K, Crociani CM (2019). Expanded Prostate Cancer Index Composite-26 (EPIC-26) Online: validation of an internet-based instrument for assessment of health-related quality of life after treatment for localized prostate cancer. Urology.

[17] Litwin MS, Lubeck DP, Henning JM, Carroll PR (1998). Differences in urologist and patient assessments of health related quality of life in men with prostate cancer: results of the CaPSURE database. J Urol.

[18] Wilkins A, Naismith O, Brand D, Fernandez K, Hall E, Dearnaley D (2020). Derivation of dose/volume constraints for the anorectum from clinician- and patient-reported outcomes in the CHHiP trial of radiation therapy fractionation. Int J Radiat Oncol Biol Phys.

[19] Andreyev HJ (2007). Gastrointestinal problems after pelvic radiotherapy: the past, the present and the future. Clin Oncol (R Coll Radiol).

[20] O'Brien PC, Hamilton CS, Denham JW, Gourlay R, Franklin CI (2004). Spontaneous improvement in late rectal mucosal changes after radiotherapy for prostate cancer. Int J Radiat Oncol Biol Phys.

[21] Gomez L, Andres C, Ruiz A (2017). Dosimetric impact in the dose-volume histograms of rectal and vesical wall contouring in prostate cancer IMRT treatments. Rep Pract Oncol Radiother.

[22] Bruner DW, Hunt D, Michalski JM, Bosch WR, Galvin JM, Amin M (2015). Preliminary patient-reported outcomes analysis of 3-dimensional radiation therapy versus intensity-modulated radiation therapy on the high-dose arm of the Radiation Therapy Oncology Group (RTOG) 0126 prostate cancer trial. Cancer.

[23] Szymanski KM, Wei JT, Dunn RL, Sanda MG (2010). Development and validation of an abbreviated version of the expanded prostate cancer index composite instrument for measuring health-related quality of life among prostate cancer survivors. Urology.

[24] Yeung AR, Pugh SL, Klopp AH, Gil KM, Wenzel L, Westin SN (2020). Improvement in patient-reported outcomes with intensity-modulated radiotherapy (RT) compared with standard RT: a report from the NRG oncology RTOG 1203 study. J Clin Oncol.

[25] Malone S, Croke J, Roustan-Delatour N, Belanger E, Avruch L, Malone C (2012). Postoperative radiotherapy for prostate cancer: a comparison of four consensus guidelines and dosimetric evaluation of 3D-CRT versus tomotherapy IMRT. Int J Radiat Oncol Biol Phys.

[26] Bell LJ, Cox J, Eade T, Rinks M, Kneebone A (2014). The impact of rectal and bladder variability on target coverage during post-prostatectomy intensity modulated radiotherapy. Radiother Oncol.

[27] Bell LJ, Cox J, Eade T, Rinks M, Kneebone A (2013). Prostate bed motion may cause geographic miss in post-prostatectomy image-guided intensity-modulated radiotherapy. J Med Imaging Radiat Oncol.

[28] Akin M, Oksuz DC, Iktueren B, Ambarcioglu P, Karacam S, Koca S (2014). Does rectum and bladder dose vary during the course of image-guided radiotherapy in the postprostatectomy setting?. Tumori.

[29] Chao M, Ho H, Joon DL, Chan Y, Spencer S, Ng M (2019). The use of tissue fiducial markers in improving the accuracy of post-prostatectomy radiotherapy. Radiat Oncol J.

[30] Gawish A, Chughtai AA, Eble MJ (2018). Dosimetric and volumetric effects in clinical target volume and organs at risk during postprostatectomy radiotherapy. Strahlenther Onkol.

[31] Elakshar S, Tsui JMG, Kucharczyk MJ, Tomic N, Fawaz ZS, Bahoric B (2019). Does interfraction cone beam computed tomography improve target localization in prostate bed radiotherapy?. Technol Cancer Res Treat.

